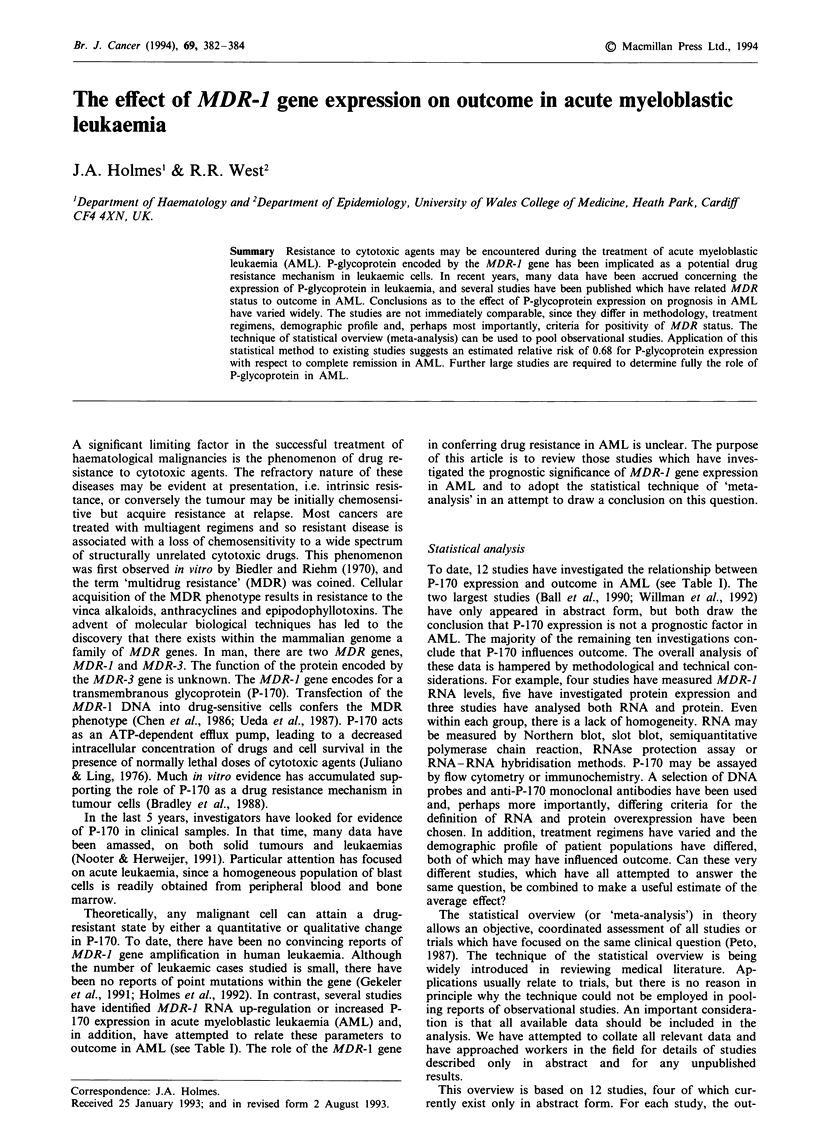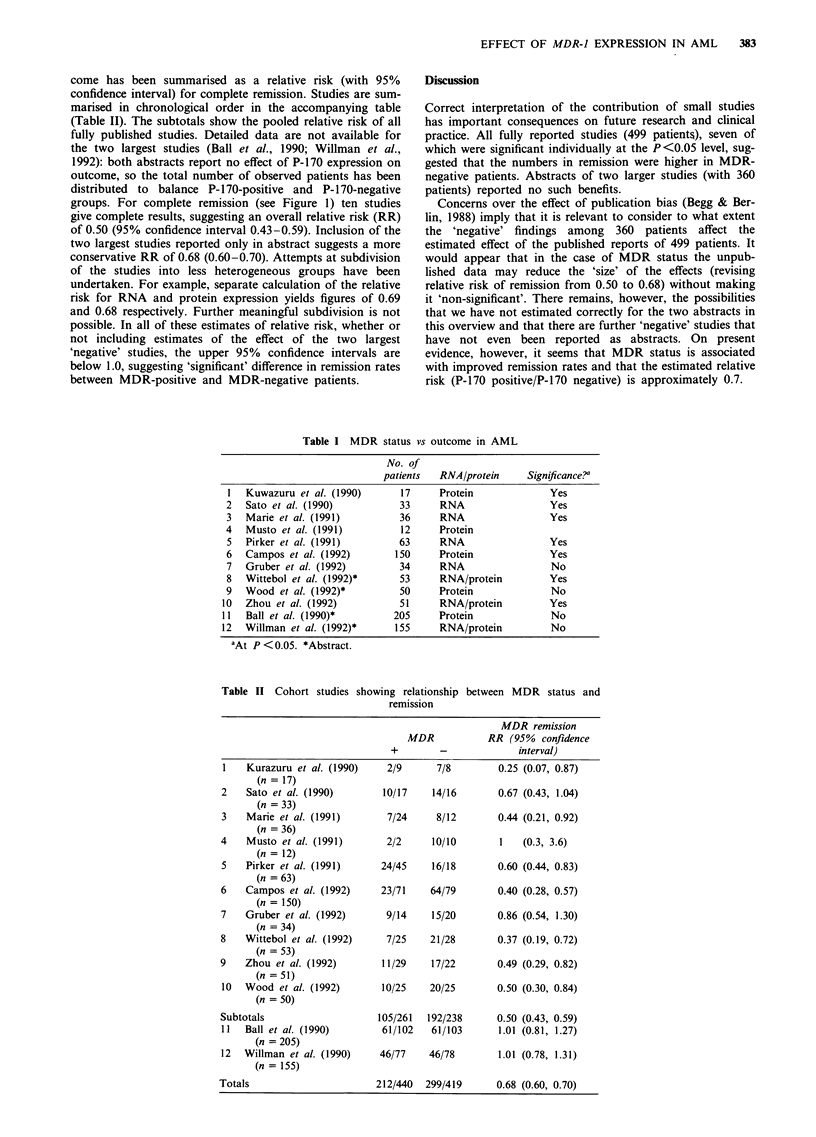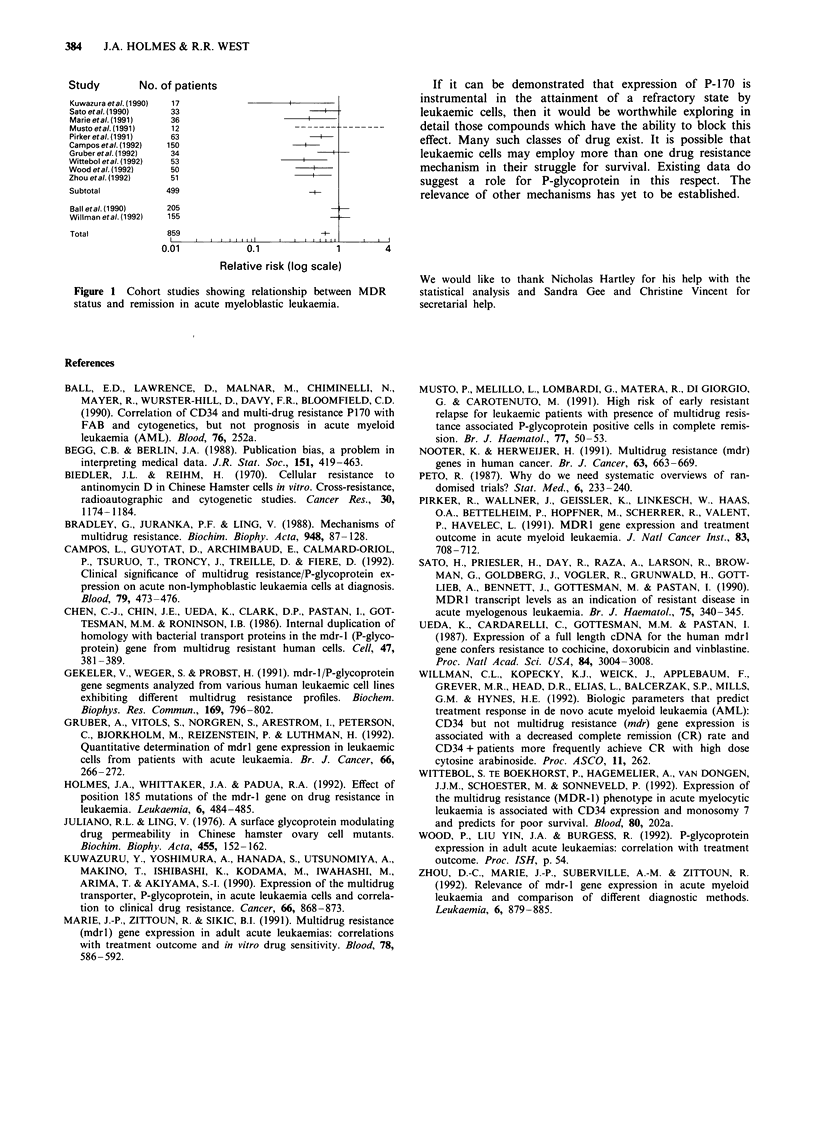# The effect of MDR-1 gene expression on outcome in acute myeloblastic leukaemia.

**DOI:** 10.1038/bjc.1994.70

**Published:** 1994-02

**Authors:** J. A. Holmes, R. R. West

**Affiliations:** Department of Haematology, University of Wales College of Medicine, Heath Park, Cardiff, UK.

## Abstract

Resistance to cytotoxic agents may be encountered during the treatment of acute myeloblastic leukaemia (AML). P-glycoprotein encoded by the MDR-1 gene has been implicated as a potential drug resistance mechanism in leukaemic cells. In recent years, many data have been accrued concerning the expression of P-glycoprotein in leukaemia, and several studies have been published which have related MDR status to outcome in AML. Conclusions as to the effect of P-glycoprotein expression on prognosis in AML have varied widely. The studies are not immediately comparable, since they differ in methodology, treatment regimens, demographic profile and, perhaps most importantly, criteria for positivity of MDR status. The technique of statistical overview (meta-analysis) can be used to pool observational studies. Application of this statistical method to existing studies suggests an estimated relative risk of 0.68 for P-glycoprotein expression with respect to complete remission in AML. Further large studies are required to determine fully the role of P-glycoprotein in AML.


Br. J. Cancer (1994), 69, 382–384 © Macmillan Press Ltd., 1994

### The effect of *MDR-1* gene expression on outcome in acute myeloblastic leukaemia

J.A. Holmes[1] & R.R. West[2]

[1]*Department of Haematology and* [2]*Department of Epidemiology, University of Wales College of Medicine, Heath Park, Cardiff CF4 4XN, UK.*

**Summary** Resistance to cytotoxic agents may be encountered during the treatment of acute myeloblastic leukaemia (AML). P-glycoprotein encoded by the *MDR-1* gene has been implicated as a potential drug resistance mechanism in leukaemic cells. In recent years, many data have been accrued concerning the expression of P-glycoprotein in leukaemia, and several studies have been published which have related *MDR* status to outcome in AML. Conclusions as to the effect of P-glycoprotein expression on prognosis in AML have varied widely. The studies are not immediately comparable, since they differ in methodology, treatment regimens, demographic profile and, perhaps most importantly, criteria for positivity of *MDR* status. The technique of statistical overview (meta-analysis) can be used to pool observational studies. Application of this statistical method to existing studies suggests an estimated relative risk of 0.68 for P-glycoprotein expression with respect to complete remission in AML. Further large studies are required to determine fully the role of P-glycoprotein in AML.

A significant limiting factor in the successful treatment of haematological malignancies is the phenomenon of drug resistance to cytotoxic agents. The refractory nature of these diseases may be evident at presentation, i.e. intrinsic resistance, or conversely the tumour may be initially chemosensitive but acquire resistance at relapse. Most cancers are treated with multiagent regimens and so resistant disease is associated with a loss of chemosensitivity to a wide spectrum of structurally unrelated cytotoxic drugs. This phenomenon was first observed *in vitro* by Biedler and Riehm (1970), and the term 'multidrug resistance' (MDR) was coined. Cellular acquisition of the MDR phenotype results in resistance to the vinca alkaloids, anthracyclines and epipodophyllotoxins. The advent of molecular biological techniques has led to the discovery that there exists within the mammalian genome a family of *MDR* genes. In man, there are two *MDR* genes, *MDR-1* and *MDR-3*. The function of the protein encoded by the *MDR-3* gene is unknown. The *MDR-1* gene encodes for a transmembranous glycoprotein (P-170). Transfection of the *MDR*-1 DNA into drug-sensitive cells confers the MDR phenotype (Chen *et al.*, 1986; Ueda *et al.*, 1987). P-170 acts as an ATP-dependent efflux pump, leading to a decreased intracellular concentration of drugs and cell survival in the presence of normally lethal doses of cytotoxic agents (Juliano & Ling, 1976). Much *in vitro* evidence has accumulated supporting the role of P-170 as a drug resistance mechanism in tumour cells (Bradley *et al.*, 1988).

In the last 5 years, investigators have looked for evidence of P-170 in clinical samples. In that time, many data have been amassed, on both solid tumours and leukaemias (Nooter & Herweijer, 1991). Particular attention has focused on acute leukaemia, since a homogeneous population of blast cells is readily obtained from peripheral blood and bone marrow.

Theoretically, any malignant cell can attain a drug-resistant state by either a quantitative or qualitative change in P-170. To date, there have been no convincing reports of *MDR-1* gene amplification in human leukaemia. Although the number of leukaemic cases studied is small, there have been no reports of point mutations within the gene (Gekeler *et al.*, 1991; Holmes *et al.*, 1992). In contrast, several studies have identified *MDR-1* RNA up-regulation or increased P-170 expression in acute myeloblastic leukaemia (AML) and, in addition, have attempted to relate these parameters to outcome in AML (see Table I). The role of the *MDR*-1 gene in conferring drug resistance in AML is unclear. The purpose of this article is to review those studies which have investigated the prognostic significance of *MDR-1* gene expression in AML and to adopt the statistical technique of 'meta-analysis' in an attempt to draw a conclusion on this question.

Correspondence: J.A. Holmes.
Received 25 January 1993; and in revised form 2 August 1993.

### *Statistical analysis*

To date, 12 studies have investigated the relationship between P-170 expression and outcome in AML (see Table I). The two largest studies (Ball *et al.*, 1990; Willman *et al.*, 1992) have only appeared in abstract form, but both draw the conclusion that P-170 expression is not a prognostic factor in AML. The majority of the remaining ten investigations conclude that P-170 influences outcome. The overall analysis of these data is hampered by methodological and technical considerations. For example, four studies have measured *MDR-1* RNA levels, five have investigated protein expression and three studies have analysed both RNA and protein. Even within each group, there is a lack of homogeneity. RNA may be measured by Northern blot, slot blot, semiquantitative polymerase chain reaction, RNAse protection assay or RNA–RNA hybridisation methods. P-170 may be assayed by flow cytometry or immunochemistry. A selection of DNA probes and anti-P-170 monoclonal antibodies have been used and, perhaps more importantly, differing criteria for the definition of RNA and protein overexpression have been chosen. In addition, treatment regimens have varied and the demographic profile of patient populations have differed, both of which may have influenced outcome. Can these very different studies, which have all attempted to answer the same question, be combined to make a useful estimate of the average effect?

The statistical overview (or 'meta-analysis') in theory allows an objective, coordinated assessment of all studies or trials which have focused on the same clinical question (Peto, 1987). The technique of the statistical overview is being widely introduced in reviewing medical literature. Applications usually relate to trials, but there is no reason in principle why the technique could not be employed in pooling reports of observational studies. An important consideration is that all available data should be included in the analysis. We have attempted to collate all relevant data and have approached workers in the field for details of studies described only in abstract and for any unpublished results.

This overview is based on 12 studies, four of which currently exist only in abstract form. For each study, the outcome has been summarised as a relative risk (with 95% confidence interval) for complete remission. Studies are summarised in chronological order in the accompanying table (Table II). The subtotals show the pooled relative risk of all fully published studies. Detailed data are not available for the two largest studies (Ball *et al.*, 1990; Willman *et al.*, 1992): both abstracts report no effect of P-170 expression on outcome, so the total number of observed patients has been distributed to balance P-170-positive and P-170-negative groups. For complete remission (see Figure 1) ten studies give complete results, suggesting an overall relative risk (RR) of 0.50 (95% confidence interval 0.43–0.59). Inclusion of the two largest studies reported only in abstract suggests a more conservative RR of 0.68 (0.60–0.70). Attempts at subdivision of the studies into less heterogeneous groups have been undertaken. For example, separate calculation of the relative risk for RNA and protein expression yields figures of 0.69 and 0.68 respectively. Further meaningful subdivision is not possible. In all of these estimates of relative risk, whether or not including estimates of the effect of the two largest 'negative' studies, the upper 95% confidence intervals are below 1.0, suggesting 'significant' difference in remission rates between MDR-positive and MDR-negative patients.

### Discussion

Correct interpretation of the contribution of small studies has important consequences on future research and clinical practice. All fully reported studies (499 patients), seven of which were significant individually at the $P<0.05$ level, suggested that the numbers in remission were higher in MDR-negative patients. Abstracts of two larger studies (with 360 patients) reported no such benefits.

Concerns over the effect of publication bias (Begg & Berlin, 1988) imply that it is relevant to consider to what extent the 'negative' findings among 360 patients affect the estimated effect of the published reports of 499 patients. It would appear that in the case of MDR status the unpublished data may reduce the 'size' of the effects (revising relative risk of remission from 0.50 to 0.68) without making it 'non-significant'. There remains, however, the possibilities that we have not estimated correctly for the two abstracts in this overview and that there are further 'negative' studies that have not even been reported as abstracts. On present evidence, however, it seems that MDR status is associated with improved remission rates and that the estimated relative risk (P-170 positive/P-170 negative) is approximately 0.7.

**Table I** MDR status *vs* outcome in AML

| | | *No. of patients* | *RNA/protein* | *Significance?*[a] |
|---|---|---|---|---|
| 1 | Kuwazuru *et al.* (1990) | 17 | Protein | Yes |
| 2 | Sato *et al.* (1990) | 33 | RNA | Yes |
| 3 | Marie *et al.* (1991) | 36 | RNA | Yes |
| 4 | Musto *et al.* (1991) | 12 | Protein | |
| 5 | Pirker *et al.* (1991) | 63 | RNA | Yes |
| 6 | Campos *et al.* (1992) | 150 | Protein | Yes |
| 7 | Gruber *et al.* (1992) | 34 | RNA | No |
| 8 | Wittebol *et al.* (1992)* | 53 | RNA/protein | Yes |
| 9 | Wood *et al.* (1992)* | 50 | Protein | No |
| 10 | Zhou *et al.* (1992) | 51 | RNA/protein | Yes |
| 11 | Ball *et al.* (1990)* | 205 | Protein | No |
| 12 | Willman *et al.* (1992)* | 155 | RNA/protein | No |

[a]At $P<0.05$. *Abstract.

**Table II** Cohort studies showing relationship between MDR status and remission

| | | *MDR* + | *MDR* − | *MDR remission RR (95% confidence interval)* |
|---|---|---|---|---|
| 1 | Kurazuru *et al.* (1990) ($n$ = 17) | 2/9 | 7/8 | 0.25 (0.07, 0.87) |
| 2 | Sato *et al.* (1990) ($n$ = 33) | 10/17 | 14/16 | 0.67 (0.43, 1.04) |
| 3 | Marie *et al.* (1991) ($n$ = 36) | 7/24 | 8/12 | 0.44 (0.21, 0.92) |
| 4 | Musto *et al.* (1991) ($n$ = 12) | 2/2 | 10/10 | 1 (0.3, 3.6) |
| 5 | Pirker *et al.* (1991) ($n$ = 63) | 24/45 | 16/18 | 0.60 (0.44, 0.83) |
| 6 | Campos *et al.* (1992) ($n$ = 150) | 23/71 | 64/79 | 0.40 (0.28, 0.57) |
| 7 | Gruber *et al.* (1992) ($n$ = 34) | 9/14 | 15/20 | 0.86 (0.54, 1.30) |
| 8 | Wittebol *et al.* (1992) ($n$ = 53) | 7/25 | 21/28 | 0.37 (0.19, 0.72) |
| 9 | Zhou *et al.* (1992) ($n$ = 51) | 11/29 | 17/22 | 0.49 (0.29, 0.82) |
| 10 | Wood *et al.* (1992) ($n$ = 50) | 10/25 | 20/25 | 0.50 (0.30, 0.84) |
| Subtotals | | 105/261 | 192/238 | 0.50 (0.43, 0.59) |
| 11 | Ball *et al.* (1990) ($n$ = 205) | 61/102 | 61/103 | 1.01 (0.81, 1.27) |
| 12 | Willman *et al.* (1990) ($n$ = 155) | 46/77 | 46/78 | 1.01 (0.78, 1.31) |
| Totals | | 212/440 | 299/419 | 0.68 (0.60, 0.70) |

**Figure 1** Cohort studies showing relationship between MDR status and remission in acute myeloblastic leukaemia.

If it can be demonstrated that expression of P-170 is instrumental in the attainment of a refractory state by leukaemic cells, then it would be worthwhile exploring in detail those compounds which have the ability to block this effect. Many such classes of drug exist. It is possible that leukaemic cells may employ more than one drug resistance mechanism in their struggle for survival. Existing data do suggest a role for P-glycoprotein in this respect. The relevance of other mechanisms has yet to be established.

We would like to thank Nicholas Hartley for his help with the statistical analysis and Sandra Gee and Christine Vincent for secretarial help.

### References

BALL, E.D., LAWRENCE, D., MALNAR, M., CHIMINELLI, N., MAYER, R., WURSTER-HILL, D., DAVY, F.R., BLOOMFIELD, C.D. (1990). Correlation of CD34 and multi-drug resistance P170 with FAB and cytogenetics, but not prognosis in acute myeloid leukaemia (AML). *Blood*, **76**, 252a.

BEGG, C.B. & BERLIN, J.A. (1988). Publication bias, a problem in interpreting medical data. *J.R. Stat. Soc.*, **151**, 419-463.

BIEDLER, J.L. & REIHM, H. (1970). Cellular resistance to antinomycin D in Chinese Hamster cells *in vitro*. Cross-resistance, radioautographic and cytogenetic studies. *Cancer Res.*, **30**, 1174-1184.

BRADLEY, G., JURANKA, P.F. & LING, V. (1988). Mechanisms of multidrug resistance. *Biochim. Biophy. Acta*, **948**, 87-128.

CAMPOS, L., GUYOTAT, D., ARCHIMBAUD, E., CALMARD-ORIOL, P., TSURUO, T., TRONCY, J., TREILLE, D. & FIERE, D. (1992). Clinical significance of multidrug resistance/P-glycoprotein expression on acute non-lymphoblastic leukaemia cells at diagnosis. *Blood*, **79**, 473-476.

CHEN, C.-J., CHIN, J.E., UEDA, K., CLARK, D.P., PASTAN, I., GOTTESMAN, M.M. & RONINSON, I.B. (1986). Internal duplication of homology with bacterial transport proteins in the mdr-1 (P-glycoprotein) gene from multidrug resistant human cells. *Cell*, **47**, 381-389.

GEKELER, V., WEGER, S. & PROBST, H. (1991). mdr-1/P-glycoprotein gene segments analyzed from various human leukaemic cell lines exhibiting different multidrug resistance profiles. *Biochem. Biophys. Res. Commun.*, **169**, 796-802.

GRUBER, A., VITOLS, S., NORGREN, S., ARESTROM, I., PETERSON, C., BJORKHOLM, M., REIZENSTEIN, P. & LUTHMAN, H. (1992). Quantitative determination of mdr1 gene expression in leukaemic cells from patients with acute leukaemia. *Br. J. Cancer*, **66**, 266-272.

HOLMES, J.A., WHITTAKER, J.A. & PADUA, R.A. (1992). Effect of position 185 mutations of the mdr-1 gene on drug resistance in leukaemia. *Leukaemia*, **6**, 484-485.

JULIANO, R.L. & LING, V. (1976). A surface glycoprotein modulating drug permeability in Chinese hamster ovary cell mutants. *Biochim. Biophy. Acta*, **455**, 152-162.

KUWAZURU, Y., YOSHIMURA, A., HANADA, S., UTSUNOMIYA, A., MAKINO, T., ISHIBASHI, K., KODAMA, M., IWAHASHI, M., ARIMA, T. & AKIYAMA, S.-I. (1990). Expression of the multidrug transporter, P-glycoprotein, in acute leukaemia cells and correlation to clinical drug resistance. *Cancer*, **66**, 868-873.

MARIE, J.-P., ZITTOUN, R. & SIKIC, B.I. (1991). Multidrug resistance (mdr1) gene expression in adult acute leukaemias: correlations with treatment outcome and *in vitro* drug sensitivity. *Blood*, **78**, 586-592.

MUSTO, P., MELILLO, L., LOMBARDI, G., MATERA, R., DI GIORGIO, G. & CAROTENUTO, M. (1991). High risk of early resistant relapse for leukaemic patients with presence of multidrug resistance associated P-glycoprotein positive cells in complete remission. *Br. J. Haematol.*, **77**, 50-53.

NOOTER, K. & HERWEIJER, H. (1991). Multidrug resistance (mdr) genes in human cancer. *Br. J. Cancer*, **63**, 663-669.

PETO, R. (1987). Why do we need systematic overviews of randomised trials? *Stat. Med.*, **6**, 233-240.

PIRKER, R., WALLNER, J., GEISSLER, K., LINKESCH, W., HAAS, O.A., BETTELHEIM, P., HOPFNER, M., SCHERRER, R., VALENT, P., HAVELEC, L. (1991). MDR1 gene expression and treatment outcome in acute myeloid leukaemia. *J. Natl Cancer Inst.*, **83**, 708-712.

SATO, H., PRIESLER, H., DAY, R., RAZA, A., LARSON, R., BROWMAN, G., GOLDBERG, J., VOGLER, R., GRUNWALD, H., GOTTLIEB, A., BENNETT, J., GOTTESMAN, M. & PASTAN, I. (1990). MDR1 transcript levels as an indication of resistant disease in acute myelogenous leukaemia. *Br. J. Haematol.*, **75**, 340-345.

UEDA, K., CARDARELLI, C., GOTTESMAN, M.M. & PASTAN, I. (1987). Expression of a full length cDNA for the human mdr1 gene confers resistance to cochicine, doxorubicin and vinblastine. *Proc. Natl Acad. Sci. USA*, **84**, 3004-3008.

WILLMAN, C.L., KOPECKY, K.J., WEICK, J., APPLEBAUM, F., GREVER, M.R., HEAD, D.R., ELIAS, L., BALCERZAK, S.P., MILLS, G.M. & HYNES, H.E. (1992). Biologic parameters that predict treatment response in de novo acute myeloid leukaemia (AML): CD34 but not multidrug resistance (*mdr*) gene expression is associated with a decreased complete remission (CR) rate and CD34 + patients more frequently achieve CR with high dose cytosine arabinoside. *Proc. ASCO*, **11**, 262.

WITTEBOL, S. TE BOEKHORST, P., HAGEMELIER, A., VAN DONGEN, J.J.M., SCHOESTER, M. & SONNEVELD, P. (1992). Expression of the multidrug resistance (MDR-1) phenotype in acute myelocytic leukaemia is associated with CD34 expression and monosomy 7 and predicts for poor survival. *Blood*, **80**, 202a.

WOOD, P., LIU YIN, J.A. & BURGESS, R. (1992). P-glycoprotein expression in adult acute leukaemias: correlation with treatment outcome. *Proc. ISH*, p. 54.

ZHOU, D.-C., MARIE, J.-P., SUBERVILLE, A.-M. & ZITTOUN, R. (1992). Relevance of mdr-1 gene expression in acute myeloid leukaemia and comparison of different diagnostic methods. *Leukaemia*, **6**, 879-885.